# The Psoriatic Arthritis Experience in Saudi Arabia from the Rheumatologist and Patient Perspectives

**DOI:** 10.2174/1573397119666230516162221

**Published:** 2023-08-03

**Authors:** Ibrahim Alhomood, Mohamed Fatani, Mohamed Bedaiwi, Sahar Al Natour, Alper Erdogan, Aya Alsharafi, Suzan Attar

**Affiliations:** 1 Medical Specialities Department, King Fahad Medical City (KFMC), P.O. Box 59046, Riyadh, 11525, Kingdom of Saudi Arabia;; 2 Department of Dermatology, Heraa Hospital, Al Madinah Al Munawarah Rd, Mecca, Makkah, 24227, Kingdom of Saudi Arabia;; 3 Department of Medicine, Rheumatology Unit, College of Medicine, King Saud University, P.O. Box 14511, Riyadh, 11451, Kingdom of Saudi Arabia;; 4 Department of Dermatology, Imam Abdulrahman Bin Faisal University, P.O. Box 1982, Dammam, 34212, Kingdom of Saudi Arabia;; 5 Department of Medical Neurology, Eli Lilly and Company, Ulaya Dist., Riyadh, Riyadh Province, Kingdom of Saudi Arabia;; 6 Department of Rheumatology, King Abdulaziz University, P.O. Box 80200, Jeddah, 21589, Kingdom of Saudi Arabia

**Keywords:** Psoriatic arthritis, patient perspectives, disease management, rheumatologists, survey, PsA treatment

## Abstract

**Background:**

Psoriatic arthritis (PsA) is a musculoskeletal disease that adversely affects physical mobility and quality of life. It is challenging to manage because of the heterogeneous nature of the symptoms and the current treatment options.

**Purpose:**

To explore the patient and rheumatologist perspectives of PsA to help improve understanding of the disease experience and improve disease management.

**Methods:**

A descriptive, observational cross-sectional study of Saudi Arabian dermatologists and rheumatologists and patients with psoriasis or PsA was conducted. Questionnaire data were collected from 31 dermatologists, 34 rheumatologists, 90 patients with psoriasis, and 98 patients with PsA and analysed using descriptive statistics. Here, data from rheumatologists and patients with PsA are presented.

**Results:**

The results revealed similarities and differences in the rheumatologist and patient perspectives of PsA. Rheumatologists and patients agreed on the impact that PsA had on patients’ quality of life and that more education was needed. However, they differed on several aspects of disease management. Rheumatologists estimated the time to diagnosis as four times shorter than what patients experienced. Patients accepted their diagnosis more than rheumatologists perceived them to; rheumatologists perceived patients to be worried or fearful. Patients perceived joint pain as their most severe symptom, in contrast to rheumatologists, who presumed skin appearance was the most severe symptom. Reported input into PsA treatment goals differed significantly. More than half of the rheumatologists reported equal patient-physician input into goal development as opposed to <10% of patients reporting the same. Almost half of patients reported no input into the development of their treatment goals.

**Conclusion:**

The management of PsA could benefit from enhanced screening and re-evaluation of what PsA outcomes have the most value to patients and rheumatologists. A multidisciplinary approach is recommended with increased patient involvement in disease management and individualized treatment options.

## 1 INTRODUCTION

Psoriatic arthritis (PsA) is a chronic autoimmune musculoskeletal disease affecting the skin and joints. It is associated with multiple physical comorbidities, including osteoporosis, metabolic syndrome, and cardiovascular disease [1] and psychological effects such as anxiety and depression [2, 3]. PsA affects one-third of patients with psoriasis [4], and 85% of those with PsA report a history of psoriasis [5]. PsA impacts physical functioning, daily activities, emotional well-being, and health-related quality of life (QoL) [6]. Although the most impactful symptoms include pain, stiffness, swelling, fatigue, sleep disturbances, and physical disability [7-9], they often go untreated because of diagnosis delays and barriers to accessing care [6, 10].

The diagnosis of PsA is challenging for the rheumatologist because of the subtle manifestation of symptoms and the non-specific nature of its presentation, unlike the prominent features of joint arthritis [11]. Early detection is imperative yet elusive [12]. The difficulty arises in selecting the safest and most effective treatment while setting realistic treatment expectations and managing side effects [5, 13, 14]. The complexity of PsA management necessitates patient-specific therapies and individualized treatment goals [15-18]. Although disease management is targeted at preventing the progression of PsA and inflammatory damage [8], treatments often have adverse effects, leading to compliance and adherence issues for patients [19].

PsA is poorly understood and under-recognized in the Middle East because of these factors and the paucity of quality research [15]. This article describes the results of a quantitative questionnaire administered to patients with PsA and rheumatologists who treat PsA in Saudi Arabia. The objectives of this study were to explore the rheumatologist and patient perspectives of PsA, including disease management, diagnosis, treatment, referral, priorities, experience, expectations, and unmet needs, and to identify the barriers to and define the gaps in the optimal management of PsA from a rheumatologist and patient perspective.

## 2 MATERIALS AND METHODS

### 2.1 Study Design and Participants

This was a descriptive observational cross-sectional study conducted in Saudi Arabia. A sample of 34 rheumatologists, 31 dermatologists, 90 patients with psoriasis, and 98 patients with PsA consented to participate in the research. Physicians were recruited from a target list. Patients were recruited from treating physician offices. Patient participants were screened for the inclusion criterion of an existing diagnosis of PsA. Before participating in the study, all subjects signed a consent form outlining the research conditions. This manuscript presents the outcomes of the questionnaires completed by rheumatologists and patients with PsA. Data collected from dermatologists and patients with psoriasis will be reported separately.

### 2.2 Data Source

Data collection occurred from July to November 2020 for rheumatologists and from February to June 2021 for patients with PsA. The surveys were co-designed by clinical experts from Eli Lilly and a steering committee of expert rheumatologists and dermatologists. IQVIA was contracted to recruit participants, conduct all interviews, and collect and report data in compliance with ethical principles. Rheumatologist interviews included 24 questions completed over 30 minutes (Supplementary Materials: Questionnaire for Rheumatologist). Rheumatologists answered screening questions on practice location and volume of patients and survey questions that inquired about multidisciplinary approaches, patient volumes, diagnosis, treatments, treatment goal setting, and symptom/disease management practices. Patients were interviewed using 26 questions over 15 minutes (Supplementary Materials: Questionnaire for Patients with Psoriatic Arthritis). Patients with PsA answered demographic screening questions and survey questions on disease course, diagnosis, symptom and disease burden, disease management, and disease-related needs/goals and expectations.

### 2.3 Data Analysis

Descriptive analyses were used; no inferences were made. Categorical data were presented as percentages of participants; ordinal data were presented as percentage scores for each category and top 2 box (T2B) percentages for ease of comparison. T2B scores combine the proportions of respondents who have selected the two highest possible Likert scale survey responses into a single number.

### 2.4 Ethics

The study was conducted according to the Declaration of Helsinki as revised in Brazil in 2013 and was approved by the Institutional Review Board at King Fahad Medical City, Riyadh (IRB Log Number 21-192).

## 3 RESULTS

### 3.1 Demographic and Clinical Characteristics

Demographic data captured in the survey of patients with PsA indicated that they were mostly of Saudi nationality, had a college degree, never smoked, and worked full-time. None lived alone, and the average age was 47 years (Table **1**). Almost 25% of these patients were previously treated for co-morbidities that included diabetes or hypertension, and approximately 15% for thyroid disease, osteoporosis, or asthma. Less than half of the patients had not been treated for underlying conditions (Table **1**).

Survey data collected from rheumatologists indicated that more than 71% of their time was spent in public hospitals, with the remaining in privately funded settings. At any one time, these rheumatologists actively managed an average of 28 patients with PsA (range 5-120) and had a monthly caseload of approximately 275 patients regardless of their condition, of which an average of 16 were being treated for PsA (Table **1**).

### 3.2 Perspectives on PsA Referral and Diagnosis

Rheumatologists reported that it was their belief that approximately 30% of patients with PsA remain undiagnosed. Their reasons for this included a lack of patient awareness (85%), primary care awareness (59%), regular screening programs for patients with psoriasis (59%), dermatologist awareness (53%), and access to specialty medicine (47%). Only 53% of rheumatologists felt that all patients with suspected PsA were referred to them. Referrals to rheumatologists originated primarily from a dermatologist (44%).

Almost all patients reported (89%) seeing a physician within 4 months of their first non-skin-related symptom, and 75% were referred to a rheumatologist within 6 months of reporting these symptoms. Similarly, rheumatologists noted a post-referral time to patient contact of 5 months. In total, 97% of patients with PsA were seen by a rheumatologist, with the remaining 3% seen by a dermatologist. The primary symptoms that triggered a rheumatologist referral included tendon pain/swelling/tenderness, swollen digits, and joint pain/swelling/tenderness/stiffness (all 85%). Other reasons included reduced range of motion (65%), morning stiffness/tiredness (56%), elevated erythrocyte sedimentation rate/C-reactive protein level (47%), nail changes (41%), uveitis (24%), fatigue (15%), and inflammatory bowel disease (15%).

On average, patients reported receiving a PsA diagnosis 64 months after their first psoriasis symptom (range 0-277 months), with most receiving a diagnosis between 2-4 years (30%) and 1-2 years (23%). Rheumatologists reported a shorter average time to confirm PsA diagnosis of 14 months.

When diagnosing PsA, 91% of rheumatologists looked for pain/swelling/tenderness/stiffness, 79% each for swollen digits and enthesitis, 71% for morning stiffness/stiffness after resting, and 65% for symmetric joint symptoms. Nail changes and inflammatory back pain were each pre-PsA indicators for 62% of rheumatologists, and psoriasis, uveitis, and fatigue counted to a lesser extent (53%, 38%, and 26%, respectively).

**Fig. (1):**
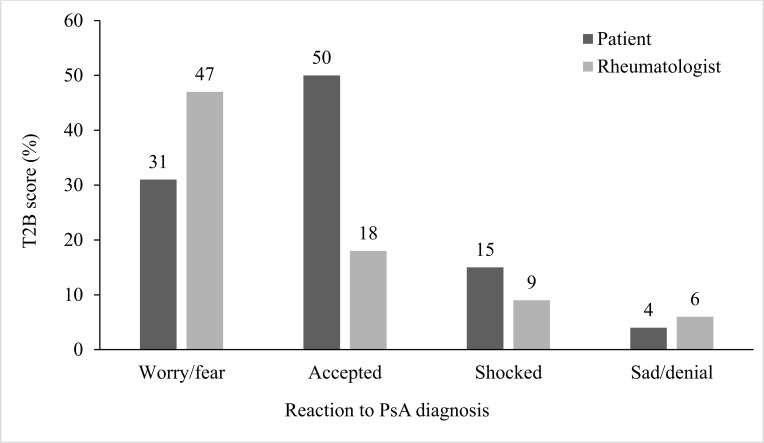
Patient and rheumatologist perspectives of the reaction to psoriatic arthritis (PsA) diagnosis. T2B, Top 2 Box.

Half of the patients responded that they had accepted their PsA diagnosis, a third were anxious/fearful, and a few were shocked or sad (Fig. **1**). In contrast, rheumatologists reported that almost half of the patients responded with worry/fear, and only a small number appeared okay, frustrated/depressed, shocked, and sad/in denial (Fig. **1**).

### 3.3 Perspectives on PsA Treatment

**Fig. (2):**
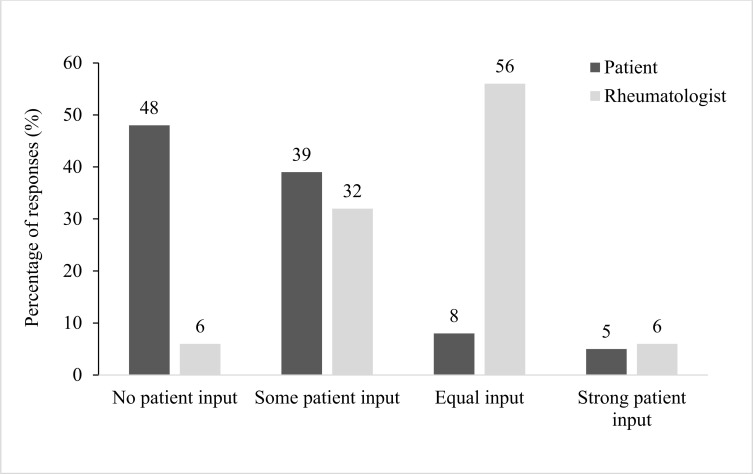
Patient and rheumatologist perspectives of input on psoriatic arthritis treatment goals.

Most patients were extremely comfortable (64%) discussing treatments with their rheumatologist. However, half of the patients reported that treatment goals were set without their input; most of the remaining patients reported some input, with only a few reporting equal or strong input (Fig. **2**). Almost half (45%) of patients noted that treatment for PsA was initiated the same day as diagnosis or within the first month. Rheumatologists responded that they shared equal input into treatment goals with their patients most of the time. Only one-third noted that they primarily set the goals, and a minority reported that patients set them or had no input (Fig. **2**).

Patients noted that follow-up visits were scheduled every 6 months (50%), every 2-3 months (44%), or every month (6%) and they were seen for 10-20 minutes (66%), less than 10 minutes (22%), or 20-30 minutes (11%). Rheumatologists reported seeing patients more frequently and for longer, with follow-up visits every month (24%), every 2-3 months (71%), or every 6 months (6%) for an average of 10-20 minutes (50%), with many visits needing 20-30 minutes (47%).

The survey results in Table **2** highlight the similarities and differences in the survey data reported by rheumatologists and patients.

### 3.4 Perspectives on PsA Symptoms and Disease Impact

**Fig. (3):**
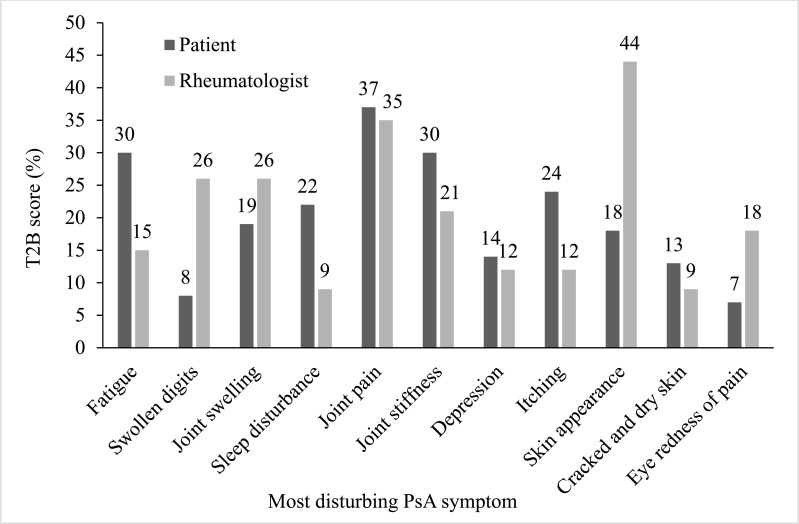
Most disturbing symptoms of psoriatic arthritis (PsA), as reported by patients and rheumatologists. T2B, Top 2 Box.

Patients reported that their most disturbing symptoms before starting treatment included joint pain, fatigue, back pain, joint stiffness in the morning, itching, and sleep disturbance (Fig. **3**). Rheumatologists viewed skin appearance as the most disturbing, with joint pain, swollen digits and joint swelling to a lesser extent (Fig. **3**). Other symptoms noted by physicians included morning stiffness, uveitis, fatigue, itchiness/burning, and depression.

The disease had the most profound impact on patients' social life, daily life, and family life (T2B: 10%, 8%, 8%, respectively). Similarly, rheumatologists agreed that PsA had the highest impact on family life, partner intimacy, and social life (T2B: 41%, 41%, 32%), respectively.

### 3.5 Perspectives on PsA Education

Although many patients reported satisfaction with the education/training they received on PsA (T2B: 37%), almost all patients (82%) would have liked more knowledge acquisition. Of these, 60% turned to social media, 45% used internet searches, and 39% used doctors for their source of information. The majority were looking for disease-related information (76%), treatment-related information (52%), and other patients’ experiences with the disease (27%). This is consistent with reports from rheumatologists in this study, who believed that only one-third (38%) of patients with PsA were well-informed and routinely questioned by their physicians about possible signs and symptoms of PsA. In addition, patients wanted more discussion with their rheumatologists on treatment convenience (36%), safety (31%), tolerability (29%), and impact on work-life (23%) and family life (21%). Only a few wanted information on treatment goals/outcomes (19%), impact on social life (18%), or their well-being (2%).

### 3.6 Patient and Rheumatologist Satisfaction

Patients were mostly satisfied with the time spent discussing treatments (T2B: 61%), the number of treatment options offered (T2B: 53%), the level of involvement (T2B: 44%), physician interaction (T2B: 47%), and education/training (T2B: 37%).

**Fig. (4):**
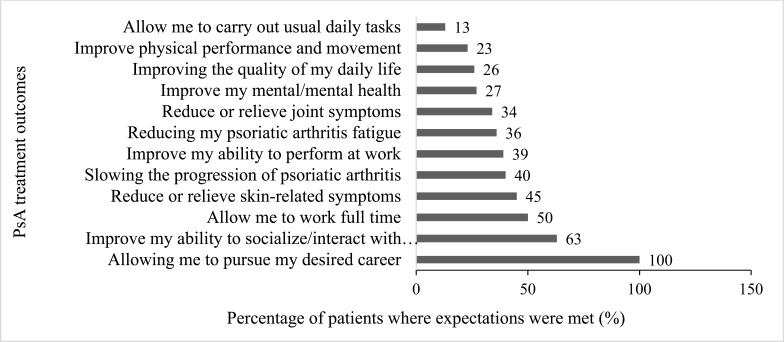
Patients’ met expectations compared with treatment outcomes. PsA, psoriatic arthritis.

Overall, patient satisfaction with PsA treatment was low (22% completely satisfied). Greater than 40% of patients reported that their initial expectation for the treatment matched the outcome of pursuing a desired career, improving social and family interactions, working full time, reducing skin-related symptoms, and slowing the progression of the disease. However, less than 30% of patient expectations were met in terms of their ability to carry out daily activities and improvements in physical movement, quality of daily life, and mental health (Fig. **4**).

The rheumatologists would have liked to have had more control over the disease with more time/resources and focus on how PsA impacts patient feelings (85%), their work life (76%), social life (68%), compliance/adherence (65%), treatment efficacy to prevent/reduce disease progression (50%), patient convenience (47%), QoL (47%), treatment safety/tolerability (44%), treatment efficacy to control symptoms (38%), and the patient’s family (32%).

## 4 DISCUSSION

This study was conducted in Saudi Arabia over 2020-2021 and explored PsA from both a rheumatologist and patient perspectives to seek ways to understand PsA better and improve disease management. Findings showed that the perspectives of rheumatologists and patients with PsA had both similarities and differences. They agreed on the time they waited to see a physician; the profound impact of PsA on family, social, and daily life; and that patients needed more education. However, their perspectives differed on the average time to receive a diagnosis, the reaction to the diagnosis, and the most disturbing PsA symptoms. They also reported significant differences in how often and for how long they received follow-up visits, their input into the treatment goals, and the content of information they would like to give/receive. Many patients highlighted differences between treatment expectations and the outcome they experienced.

Surveyed rheumatologists in this study reported that they were able to diagnose PsA within 14 months of receiving the referral. This is substantially quicker than what the surveyed patients in this study reported and what is currently found in the published literature. A population-based study conducted between 2000 and 2017 found that more than half of patients with PsA experienced a diagnostic delay of more than 24 months, with those with a higher body mass index waiting even longer [20]. Another nationwide study reported longer times to diagnosis [21], finding that, although time to diagnosis was declining, the mean time from symptom onset to PsA diagnosis was 53 months [21]. Other published studies reported wait times of 5 years between psoriasis and the development of PsA [22, 23]. This is in line with the 64 months that surveyed patients in this study reported. Of interest is that Karmacharya and colleagues [20] found that delays of even 6-12 months have been associated with poorer functional outcomes, progression of joint damage, and diminished treatment options.

The existing evidence estimates the prevalence of undiagnosed PsA to be 15.5% [4]. Rheumatologists in this study attributed high rates of undiagnosed PsA primarily to a lack of patient awareness. However, the published literature indicates that delays are likely a result of the heterogeneous presentation of PsA, the lack of a definitive diagnostic test, the concurrent presence of psoriasis [20], and the lack of clinical expertise [24, 25]. To assist physicians and patients with PsA, the use of both screening appointments [26] and screening tools is recommended [4, 27] to enhance awareness of the importance of early diagnosis [2, 21].

Patients are managing complex functional disabilities and experiencing a profound impact on their QoL by the time they receive their PsA diagnosis. It is not surprising that many newly diagnosed patients report distress and anxiety as they come to terms with their decreased QoL [24]; however, this level of distress was not apparent in this study. Half of the surveyed patients reported that they accepted their diagnosis, unlike the rheumatologists’ report of only one in five being okay with the PsA diagnosis. Of interest is that 40% of these patients were working full-time and 58% held college degrees. Other studies note that the predictors of the psychological response to PsA include the patient’s level of support and education [24] and their illness beliefs and coping strategies [28]. These factors could be seen as potential targets for treatment interventions that address PsA-related QoL.

Several studies have noted the symptom prevalence, frequency, and severity of PsA on physical function, QoL, and psychological state [6, 9, 28, 29]. This extends beyond the individual to the societal level, where PsA is associated with significant healthcare costs, unemployment, and disability [24]. Patients with PsA in this study reported that joint pain was their primary symptom of concern, whereas rheumatologists indicated that skin appearance was the main patient priority. These results should be interpreted with care as patients with PsA who are seeing a rheumatologist may be more likely to report rheumatic symptoms. The most notable differences in symptom severity perception of surveyed participants were fatigue and itching (rated high by patients) and swollen digits and sleep disturbances (rated high by rheumatologists). Published literature indicates that symptoms with the highest priority for patients include fatigue [6, 7, 30], physical function [9], pain [6], and morning joint stiffness [30]. The misalignment in this study between physician and patient assessments has been reflected in previously published studies [6, 7, 31-34]; however, the reasons for this are not well understood, highlighting the challenges associated with the management of PsA. Nowell and colleagues [30] suggested that patient-reported symptoms can be used as a tool to prioritize PsA treatments. Accurately assessing PsA symptoms that are a priority for patients has important repercussions for treatment choices, patient expectations, and health outcomes.

In this study, patients with PsA did not feel included in setting the treatment goals. Both patients and rheumatologists agreed that fewer than one in ten patients with PsA had a strong input in treatment goal setting; half of the patients reported no input at all. The absence of patient input in treatment goals is concerning and well documented in the published literature [24, 30, 35, 36]. PsA management is moving from the conventional treat-and-monitor approach to a treat-to-target approach, which is feasible in PsA and improves patient outcomes and expectations [36, 37]. Treat-to-target PsA management identifies specific target goals/outcomes that are unique to the disease and patient [38]. This requires patient involvement in setting treatment targets with the goal of minimal disease activity [36].

Many surveyed patients in this study felt their PsA treatment expectations matched the outcomes, but unmet expectations were highlighted in a few areas. These unmet needs included the inability to carry out usual activities and improvement in physical movement, QoL, and mental health. Despite treatment advances, evidence [39] suggests that a large number of patients with PsA continue to experience uncontrolled disease, do not achieve minimal disease activity, and are dissatisfied with current therapies. Similarly, only 22% of patients surveyed in this study were completely satisfied with their PsA treatments. To improve the congruence between treatment expectations and outcomes, existing outcome measures need to be refined, assessment tools standardized [8, 21], and a patient-centered approach with target goals taken [35, 37].

Limitations of this study include the study design and sample size. Cross-sectional studies cannot establish incidence or trends, only prevalence and associations between variables at a single point in time. Furthermore, the sample size in this study was small and may not be representative of the larger population. Participants' recruitment, selection, and memory recall may also have involved bias. Therefore, these findings have limited generalizability beyond the sample population and geographical area.

## CONCLUSION

The findings from this study suggest that rheumatologists and patients with PsA differ in their perspectives of PsA management, including the time and reaction to the PsA diagnosis, symptom severity, and their participation in treatment goals. This may have contributed to patient and physician dissatisfaction with PsA management and unmet expectations with treatment plans. PsA management could benefit from a multidisciplinary approach, standardized assessment and screening tools, and the re-evaluation of PsA outcomes. The inclusion of patient-focused education and patients in treatment plan development is imperative to reach the goal of minimal disease activity in PsA. This study also highlights the need for further research of rheumatologists and patient perspectives of the disease pathway from psoriasis to PsA.

## Figures and Tables

**Table 1:** Demographic characteristics of survey participants.

**Characteristic**	**N (%)**
Patients with psoriatic arthritis (n = 98)	
Mean age, years Mean height, cm Mean weight, kg	47 159 76
Nationality Saudi Arabian Expat Arab East Asian	81 (83) 15 (15) 2 (2)
Living situation Living with spouse/family Living alone	98 (100) 0
Employment status Working full-time A homemaker Retired Self-employed Working part-time	39 (40) 35 (36) 14 (14) 5 (5) 5 (5)
Education College degree Postgraduate degree High school Elementary school	57 (58) 20 (20) 17 (17) 4 (4)
Smoking status Never smoked Current smoker Past smoker	74 (76) 17 (17) 7 (7)
Conditions receiving treatment Diabetes Hypertension Thyroid disease Osteoporosis Asthma Cardiovascular disease Crohn’s disease Not currently receiving treatment for these	24 (24) 23 (23) 17 (17) 16 (16) 15 (15) 4 (4) 3 (3) 41 (42)
Rheumatologists (n = 34)	
Work setting Public hospital Private hospital/clinic	24 (71) 10 (29)
Patient volume in 1 month All patients regardless of diagnosis Patients treated for psoriatic arthritis	275 16

**Table 2:** Comparison of the perspectives of rheumatologists and patients with PsA on the psoriatic arthritis journey.

**Perspective**	**Rheumatologist (n = 34)**	**Patients with PsA (n = 98)**
Physician contact	5 months	4 months
Average time to diagnosis	14 months from referral	64 months from symptom onset
Reaction to diagnosis	Worry/fear (47%), OK (18%), frustration (15%), shocked (9%), sad/denial (6% each)	Accepted (50%), anxious/fearful (31%), shocked (15%), sad (4%)
Most disturbing symptoms	Skin appearance (T2B: 44%), joint pain (T2B: 35%), swollen digits (T2B: 26%), and joint swelling (T2B: 26%)	Joint pain (T2B: 37%), fatigue (T2B: 30%), back pain (T2B: 31%), joint stiffness in the morning (T2B: 30%, itching (T2B: 24%), and sleep disturbance (T2B: 22%)
Treatment goals	Equal input (56%), some patient input (32%), strong patient input (6%), no patient input (6%)	No patient input (48%), some patient input (39%), equal input (8%), strong patient input (5%)
Satisfaction with treatment	*	Satisfaction with PsA treatment was low (highly satisfied T2B: 40%; completely satisfied 22% total).
Follow-up	Monthly (24%), every 2-3 months (71%), or every 6 months (6%) for an average of 10-20 minutes (50%) or 20-30 minutes (47%)	Every 6 months (50%), every 2-3 months (44%), or every month (6%) for 10-20 minutes (66%), less than 10 minutes (22%), or 20-30 minutes (11%)
Disease impact on life	Profound impact on family life and partner intimacy (T2B: 41% each), social life (T2B: 32%), everyday activities (T2B: 18%), and work life (T2B: 12%)	Profound impact on social life, daily life, and family life (T2B: 10%, 8%, 8%)
Education	38% believe patients are well-informed	82% sought additional information on disease (76%), treatments (52%), and other’s disease experience (27%)
Additional discussion	More time/resources to focus on feelings/ wellbeing (85%), their work life (76%), social life (68%), compliance/adherence (65%), treatment efficacy to prevent/reduce disease progression (50%), patient convenience (47%), QoL (47%), treatment safety/tolerability (44%), treatment efficacy to control symptoms (38%), impact on family (32%)	Most discussion on treatment convenience (36%), safety (31%), tolerability (29%), and impact on work life (23%) and family life (21%) Few wanted more information on treatment goals/outcomes (19%), impact on social life (18%), or how they felt/well-being (2%)
Unmet expectations	*	The expectation for the treatment matched the result in the following areas: pursuing a desired career (100%), improving social and family interactions (T2B: 63%), working full time (T2B: 50%), reducing skin-related symptoms (T2B: 45%), and slowing the progression of disease (T2B: 40%). Areas where the expectations were not adequately met were the ability to carry out usual daily activities (T2B: 13%), improved physical movement (T2B: 23%), improved QoL (T2B: 26%), and improved mental health (T2B: 27%)

**Note:** * Question was not asked in this participant group. **Abbreviations:** PsA, psoriatic arthritis; QoL, quality of life; T2B, Top 2 Box.

## Data Availability

The data used to support the findings of this study are available from the corresponding author upon request.

## LIST OF ABBREVIATIONS

PsA: Psoriatic Arthritis
QoL: Quality of Life
T2B: Top 2 Box
