# Supplementary material for: The Psoriatic Arthritis Experience in Saudi Arabia from the Rheumatologist and Patient Perspectives
Source: Curr Rheumatol Rev. 2023 Aug 3;19(4):470–8. doi: 10.2174/1573397119666230516162221 (PMC10523353; doi:10.2174/1573397119666230516162221)
Supplement: Supplementary file 1 — Supplementary material is available on the publisher’s website along with the published article. 1. Questionnaire for Rheumatologists. 2. Questionnaire for Patients with Psoriatic Arthritis. [file CRR-19-470_SD1.pdf]

Supplementary Material

The Psoriatic Arthritis Experience in Saudi Arabia from the Rheumatologist and Patient Perspectives

Ibrahim Alhomood[1,*], Mohamed Fatani[2], Mohamed Bedaiwi[3], Sahar Al Natour[4], Alper Erdogan[5], Aya Alsharafi[5] and Suzan Attar[6]

[1]King Fahad Medical City (KFMC), P.O. Box 59046, Riyadh, 11525, Kingdom of Saudi Arabia; [2]Heraa Hospital, Al Madinah Al Munawarah Rd, Mecca, Makkah, 24227, Kingdom of Saudi Arabia; [3]Rheumatology Unit, Department of Medicine, College of Medicine, King Saud University, P.O. Box 14511, Riyadh, 11451, Kingdom of Saudi Arabia; [4]Imam Abdulrahman Bin Faisal University, P.O. Box 1982, Dammam, 34212, Kingdom of Saudi Arabia; [5]Eli Lilly and Company, Ulaya Dist., Riyadh, Ulaya Dist., Riyadh, Riyadh Province, Kingdom of Saudi Arabia; [6] King Abdulaziz University, P.O. Box 80200, Jeddah, 21589, Kingdom of Saudi Arabia

Psoriatic Arthritis Patient Journey Research

Rheumatologists Questionnaire

Questionnaire Number		
Interviewer Name	Date of interview	Supervisor name

Back Checked By:	Date of Back Check:	Result of Back Check (OK, Query, Reject)

**RESPONDENT DETAILS**	
Physician name	
Hospital/Clinic name	
Telephone number	

1875-6360/23 $65.00+.00 © 2023 Bentham Science Publishers

Screener – Rheumatologists

IMPORTANT CRITERIA
**PRIORITY CRITERIA FOR ALL RESPONDENTS:**<br>**1.** Primary medical specialty of Rheumatology<br>**2.** Treats >5 Psoriatic Arthritis patients (PsA)<br>**3.** Able and willing to complete questionnaires<br>**4.** Able and willing to help collection of data from patients

INTRODUCTION & SCOPE

Good morning/afternoon/evening. My name is ____________________, I work for IQVIA who are currently conducting an important market research. The research is being sponsored by a pharmaceutical company. The research is being conducted with physicians in the area of Psoriatic Arthritis.

First, I would like to check that you are eligible to take part in this study; may I ask you a few quick questions?

**S1 [ALL]** Could you please tell me your primary specialty?		
Rheumatologists	1	**CONTINUE TO S2**
Other	2	**CLOSE**

**S2 [ALL]** Where do you spend majority of your practice time? Please select one response only.	
Public hospital (MOH/ Institutional)	1
Privately funded hospital/ Office based clinic	2

**S3 [ALL]** Approximately, how many patients with Psoriatic Arthritis are you actively managing? Please consider unique patients, not patient visits.	
# Psoriatic Arthritis patients………	**CLOSE IF <5 Patients**

**S4 [ALL]** During the course of our study we are aiming to collect information from Psoriatic Arthritis patients about their disease experience, would you be willing to help us identify and collect the needful information from the patients for the purpose of market research?		
Yes	1	**CLOSE**
No	2	**CONTINUE**

Thank you for your interest in our study, we are inviting physicians like yourself to take part in our study. This is purely a market research exercise to help us understand more about your views and opinions. No one will attempt to sell you anything either during the interview or afterwards. We would very much appreciate your opinions.

The research will take up to 30 minutes in total. We are offering an honorarium of (insert as appropriate) to those who take part.

Would you be interested in participating in this discussion?

Yes 1 **RECRUIT**

No 2 **CLOSE**

PERSONAL DATA: Please be informed that we limit the personal data we hold about you to contact details and information about your work place and specialization in order to conduct our studies.

Your responses and any personal contact information you provide in completing the survey will be:

- Processed by the IQVIA Incorporated group of companies ("IQVIA") on a strictly need-to-know basis, for purposes of informing IQVIA and its clients of current and on-going trends in the management of Psoriatic Arthritis and for any follow-up contact that you have consented to.
- Disclosed to the sponsoring pharmaceutical company, meaning that the answers are linked to your identity, for use by the sponsoring pharmaceutical company to define its marketing strategy and orientate communications with healthcare professionals.
- Passed on to the sponsoring pharmaceutical company in the event of adverse event reporting**
- Stored securely on IQVIA servers located in the EU, US, and other countries where necessary, in accordance with applicable data protection laws and retained only as long as necessary for the purposes of use outlined herein.

**ADVERSE EVENTS REPORTING: We are now being asked to pass on to our client, details of adverse events that are mentioned during the course of market research. Should you raise, during the survey, an adverse event in a specific patient or in a specific number of patients, we will need to report this even if it has already been reported by you directly to the company or the regulatory authorities. Due to the nature of this study being at a named level, should you agree to proceed with this survey, your contact details, along with the adverse event, will be passed on to the drug safety department of the company.

CONFIDENTIALITY AGREEMENT: You acknowledge that in the course of this study, proprietary information regarding products and product development, and other trade secrets and know-how may be disclosed, and by participating in this study you agree to hold all such information confidential and to not disclose it to any third party or use it for any other purpose whatsoever. You also agree not to disclose any part of the following pages, which are proprietary material of IQVIA and its clients. You are required to accept the above confidentiality agreement in order to participate in this survey.

I confirm my agreement to proceed with this research and to the processing of my personal information provided to IQVIA, as stated above.

Accept our conditions and start now 1

Cancel 2

MAIN QUESTIONNAIRE

1. Approximately how many patients do you treat during a typical month regardless their condition?

   .... # patients seen

2. Approximately how many patients do you see for the treatment of PsA during a typical month?

   .... # Psoriatic Arthritis patients seen

3. What percentage of Psoriatic Arthritis patients you currently treat are referred to you from dermatologists?

   ………………………..%

4. What percentage of all Psoriatic Arthritis patients in KSA, do you believe are still undiagnosed?

   ………………………%   Don't know ❑

5. In your opinion, what are some of the key reasons because of which some people within the KSA population despite living with a suspected Psoriatic Arthritis condition remain undiagnosed?

Reason	Multiple Choice
• Lack of awareness among patients	❑
• Lack / Low awareness among primary care physicians	❑
• Lack / Low awareness among dermatologists	❑
• Lack of access to specialty medicine (2ry / 3ry care)	❑
• No regular screening programs for psoriasis patients	❑
Other, specify	❑

6. Thinking of PsA patients that are referred to you form dermatology, what are the key findings that trigger suspected Psoriatic Arthritis patients to be referred to a rheumatologist?

Symptoms	*Select all that apply*
Fatigue	❑
Elevated ESR or CRP	❑
Tenderness, pain and swelling over tendons	❑
Swollen fingers and toes	❑
Stiffness, pain, throbbing, swelling and tenderness in joints	❑
A reduced range of motion or disability progression	❑
Morning stiffness and tiredness	❑
Nail changes	❑
Uveitis	❑
IBD	❑

7. Do you have a practice of combined clinic with dermatologists in your hospital?

   Yes ❑   No ❑

8. What is the average time lapse between beginning of Psoriatic Arthritis symptoms and confirming the diagnosis of Psoriatic Arthritis?

Months	Years

9. What is the average timeframe between patients' first pre-diagnosis visits to their referring doctors and their first visit to you (months)?

Months

10. What are the most common signs, symptoms that you look for in a patient that prompts you to initiate diagnosis of Psoriatic Arthritis?

Symptoms	***Select all that apply***
Fatigue	❑
Presence of psoriasis	❑
Swelling, tenderness and inflammation of the joints (e.g. knee, elbow)	❑
Morning stiffness or stiffness after resting	❑
Swollen fingers and toes	❑
Asymmetric joint symptoms	❑
Enthesitis (pain and swelling of the entheses or places where tendons and ligaments connect to bone)	❑
Nail changes	❑
Inflammatory-type back pain that is worse with rest and better with activity	❑
Redness and swelling in the eye	❑
Others, specify____________________	❑

11. What is usually initial reaction of patients to diagnosis?

Reaction	Single Choice
• Okay	❑
• Worried / afraid	❑
• Sad	❑
• Frustrated / depressed	❑
• Denial	❑
• Shocked	❑

12. Thinking about psoriatic arthritis patients' life overall, how much does their disease impact upon each of the following aspects?
Please use a 10-point scale where "1" means very minor impact, and "10" means very high impact

Impact that PsA has upon patients'….	*Impact (1-10)*
Everyday activities	
Work life	
Social life	
Family life	
Intimacy and relationship with partner	

13. Thinking of your PsA patients can you rate level of your symptoms that were most disturbing? Please use a 10-point scale where "1" means not at all disturbing, and "10" means extremely disturbing

Symptoms	*Rating (1-10)*
Joint Pain	
Joint Swelling	
Swollen finger or toe	
Morning stiffness	
Skin appearance	
Itchiness / burning sensation	
Cracked dry skin	
Skin bleeding	
Burning sensation in skin	
Fatigue	
Depression	
Sleep disturbance	
Eye redness or pain	

14. When treatment goals / desired outcomes were set, how much influence did you have in setting these compared to your patient?

I had **no input** and the goals were solely **set by my patient**	❑
I had **some input,** but the goals were **mainly set by my patient**	❑
My patient and I had **equal input**	❑
I had **strong input** and the goals were **mainly set by me**	❑
I had **full input** and the goals were **solely set by me**	❑

15. How regularly do your patients visit you to review treatment success?

Weekly	❑
Monthly	❑
Every 2-3 months	❑

Every 6 months	❑
Yearly	❑
Less often than every 12 months	❑

16. How much time do you allocate to your patient during your regular visits?

Less than 10 minutes	❑
10-20 minutes	❑
20-30 minutes	❑
>30 minutes	❑

17. Which aspects of disease that you would you like to improve further for your patient if you had more time and resources?

Impact upon patient's work life	❑
Impact upon patient's social life	❑
Impact upon patient's family	❑
Impact upon how my patients feel / their mental/psychological well-being	❑
Other (please specify): ____	❑

18. What do you feel is missing or needed to improve overall success of disease management in your practice?

	**Multiple select**
Treatment efficacy in controlling disease symptoms	1
Treatment efficacy in preventing / reducing disease progression	2
Treatment safety / tolerability	3
Patients convenience (treatments route of administration, frequency)	4
Patients' compliance / adherence	5
Patients' Quality of Life whilst on treatment	6
Other, specify	7

19. What percentage of your PsA patients received biologic treatment before your intervention?

……………………….. %

20. When starting biologic treatments, which one/s you prescribe the most as 1st biologic, and which one/s prescribe the most as 2nd biologic **(you can choose maximum 2 drugs per each column)**

Treatment	1st biologic	2nd biologic
Adalimumab e.g., Humira	❑	❑
Etanercept e.g., Enbrel®	❑	❑
Infliximab e.g., Remicade®	❑	❑
Golimumab e.g., Simponi®	❑	❑
Certolizumab Pegol e.g., Cimzia®	❑	❑
Ustekinumab e.g., Stelara®	❑	❑
Secukinumab e.g., Cosentyx®	❑	❑
Ixekizumab e.g., Taltz®	❑	❑
Guselkumab e.g., Tremfya®	❑	❑
Risankizumab e.g., Skyrizi®	❑	❑
Tildrakizumab e.g., Ilumya®	❑	❑
Apremilast e.g., Otezla®	❑	❑
Tofacitinib e.g., Xeljanz®	❑	❑

21. For the treatment you prescribe most (mentioned in the previous question), please provide the reason why you chose each?

<table>
<tr><th></th><th>Insert Brand/ Generic name</th><th>Reason/s for choice (Multiple choice allowed)</th></tr>
<tr><td rowspan="2">1st biologic</td><td></td><td>• Efficacy
• Safety
• Guidelines
• Mechanism of action
• Ease of use / patient compliance
• Cost effective</td></tr>
<tr><td></td><td>• Efficacy
• Safety
• Guidelines
• Mechanism of action
• Ease of use / patient compliance
• Cost effective</td></tr>
<tr><td rowspan="2">2nd biologic</td><td></td><td>• Efficacy
• Safety
• Guidelines
• Mechanism of action
• Ease of use / patient compliance
• Cost effective</td></tr>
<tr><td></td><td>• Efficacy
• Safety
• Guidelines
• Mechanism of action
• Ease of use / patient compliance
• Cost effective</td></tr>
</table>

22. which of the below HCPs in your hospital you closely interact with for PsA management?
and how satisfied are you with this engagement? (using a 10-point scale)

	**You interact with for PsA management**	**Satisfaction (1-10)**
Dermatologist	❑	
Psychologist	❑	
GP / Family Physician	❑	
Pain Specialist	❑	
Internal Medicine	❑	
Nurse	❑	
Other type of healthcare provider (Which one/s)…………………………..	❑	

23. Do you believe all Psoriasis patients are well informed and questioned routinely on all possible signs and symptoms of PsA?

• Yes	❑
• No	❑

24. Do you believe all suspected PsA patients are referred to your department in your hospital?

• Yes	❑
• No	❑

Psoriatic Arthritis Patient Journey Research

PsA Patients Questionnaire

Questionnaire Number		
**Interviewer Name**	**Date of Interview**	**Supervisor Name**

Back Checked By:	Date of Back Check:	Result of Back Check (OK, Query, Reject)

RESPONDENT DETAILS		
Name		
Gender	Male ❑	Female ❑
Contact number (Optional)		
Record Region/city **(KSA)**		

INCLUSION CRITERIA
**PRIORITY CRITERIA FOR ALL RESPONDENTS:**<br>**5.** Confirmed PsA diagnosis by CASPAR criteria<br>**6.** Aged ≥18 years<br>**7.** Able to read, speak, and understand Arabic or English<br>**8.** Able and willing to complete questionnaires

Definition:

The CASPAR criteria consist of confirmed inflammatory articular disease (joint, spine, or entheseal) with at least 3 points from the following features:

- current psoriasis (assigned a score of 2 points; all other features are assigned a score of 1),
- a history of psoriasis or a family history of psoriasis (unless current psoriasis is present),
- a swelling of a finger (dactylitis)
- X-ray evidence of new bone growth near a joint (juxtaarticular)
- rheumatoid factor (RF) negativity (except latex test)
- psoriatic nail dystrophy.

Screener Questions

S1. Record nationality: **SINGLE CODE ONLY**

Saudi	1
Expat Arab	2
East Asian	3
South East Asian	4
Westerner	5

S2. Record age in completed years: ………..**YEARS**

S3. What is your height in Cm? _________cm

S4. What is your weight in Kg? _________kg

S5. Record Highest level of education

Education status	Single select
Elementary school	❑
High school	❑
College degree	❑
Post Graduate degree	❑

S6. Record Current living/domestic situation

Living alone	❑
Living with a spouse / family	❑

S7. Record Employment Status

Employment status	Single select
Working full time	❑
Working part time	❑
Self-employed	❑
A homemaker	❑
Retired	❑
Unable to work	❑

S8. Smoking History

Smoking status	Single select
Current smoker	❑
Past smoker	❑
Never smoked	❑

MAIN QUESTIONNAIRE

IQVIA, a global healthcare insights and market research company, is seeking your input in developing an in-depth understanding of patients' disease experience.

We are conducting this survey on behalf of a pharmaceutical company. Any information presented during the course of this research is done solely to explore reactions to such information and should be assumed to represent hypotheses about what can be said about a product or disease area. It will not be used to influence decisions outside the research setting.

Please be assured that this survey is strictly for marketing research purposes and is being carried out within the defined Market Research code of conduct for your country. The information you provide will be treated as confidential and your identity will not be revealed to a third party without your prior consent. The survey will take approximately **15 minutes** to complete. Based on this information, are you willing to proceed with the survey?

Yes	CONTINUE
No	TERMINATE

ADVERSE EVENTS

Interviewer say:

"We are required by law to pass on to our client details of any side effects or product technical complaints related to their own products that are mentioned during the course of market research.

Although what you say will, of course, be treated in confidence, should you mention during the discussion a side effect or product technical complaint when you, or someone you know, became ill after taking one of our client's products, or a problem you have had with one of our client's products we will need to report this, so that they can learn more about the safety of their products.

Are you happy to proceed with the interview on this basis?"

Yes	CONTINUE
No	TERMINATE

In case you mention an adverse event during this research, we would file a report without giving any of your details, but if the Drug Safety Department requires more information, would you be willing to waive the confidentiality given to you under the Codes of conduct specifically in relation to that adverse event, so they can contact you directly for further information?

Please note that if you provide your name during the Adverse Event reporting, this will not be linked in any way to the responses given during the program and everything else you say during the course of the interview will continue to remain confidential.

Yes	CONTINUE
No	CONTINUE

1. Which of the following conditions, if any, are you currently treated for?

Condition	Multiple select
Asthma	❑
Heart Disease or other cardiovascular condition	❑
Osteoporosis	❑
Systemic Lupus Erythematosus (SLE)	❑
Multiple Sclerosis	❑
Crohn’s Disease	❑
Ulcerative Colitis	❑
Bipolar / schizophrenia	❑
High blood pressure	❑
Diabetes	❑
Thyroid disease	❑
Depression	❑
I am not currently treated for any of these conditions	❑

2. Please tell us the year and month in which you were officially diagnosed with **psoriatic arthritis**?

Year	Month

3. (if there’s prior PsO diagnosis) Please tell us the year and month in which you were first diagnosed with **psoriasis**?

Year	Month

4. Since you started feeling your first non-skin related symptoms (like pain and swelling in your joints), how long did it take for you to talk with your physician regarding these symptoms?

	Single Select
Within a month	❑
Within 2-4 months	❑
Within 4-6 months	❑
Within 6-12 months	❑
Longer	❑

5. And then, how long did it take for your doctor refer you to a rheumatologist?

	**Single Select**
Same day as he/she learned my new symptoms	❑
Within a week	❑
Within 2-3 weeks	❑
Within a month	❑
Within 2-3 months	❑
Within 4-6 months	❑
Longer	❑
Did not refer to a rheumatologist	❑

6. Which specialty does your primary treating physician belong to?

	**Single Select**
Dermatology	❑
Rheumatology	❑
Internal medicine	❑
Family medicine	❑
Other	❑

7. Thinking about when you were first diagnosed with psoriatic arthritis how long was it before you were started on your first treatment?

	**Single Select**
Same day as diagnosis	❑
Within a week	❑
Within 2-3 weeks	❑
Within a month	❑
Within 2-3 months	❑
Within 4-6 months	❑
Within 7-12 months	❑
Within 1-2 years	❑
Longer	❑

8. What was your initial reaction & how did you accepted the fact of being Psoriatic Arthritis patient?

Reaction	**Single Select**
Okay	❑
Worried / afraid	❑
Sad	❑
Frustrated / depressed	❑
Denial	❑
Shocked	❑

9. Thinking about your life overall, how much does your disease impact upon each of the following? Using a 10-point scale where "1 = very minor impact", and "10 = very high impact"

Impact that Psoriatic Arthritis has upon your….	***Impact (1-10)***
Everyday life	
Work life	
Social life	
Family life	
Intimacy with partner	
Relationship with partner	

10. Rate level of your symptoms that was most disturbing for you before you started treatment.

Use a 10-point scale where "1" means not at all disturbing, and "10" means extremely disturbing

	Rate (1-10)
Joint Pain	
Joint Swelling	
Back pain	
Swollen finger or toe	
Morning stiffness	
Skin appearance	
Itchiness	
Cracker dry skin	
Skin Bleeding	
Burning sensation	
Fatigue	
Depression	
Sleep disturbance	
Eye redness or pain	

11. Thinking about when you started your current treatment was a plan with specific treatment goals / desired outcomes set with your physician?

• Yes	❑
• No	❑

12. What treatment goals / desired outcomes were set when you started your treatment?

Please **Rank** the top 5 goals in order of importance for you, where 1 is the most important, 2 is the next and so on

	**Multiple select**	**Rank top 5**
Reducing or alleviating my skin-related symptoms	❑	
Reducing or alleviating my joint-related symptoms	❑	
Improving my overall, everyday quality of life	❑	
Improving my ability to socialize/interact with friends and family	❑	
Allowing me to engage in exercise/fitness activities/sports	❑	
Allowing me to carry out usual, daily tasks	❑	
Improving my ability to perform at work	❑	
Improving my disease sufficiently to allow me to have a job	❑	
Allowing me to work full-time	❑	
Allowing me to pursue my desired career	❑	
Minimizing the pain I experience due to my psoriatic arthritis (taking joint and skin pain into consideration)	❑	
Minimizing the fatigue I experience from my psoriatic arthritis	❑	
Improving my physical functioning and mobility	❑	
Slowing the progression of my psoriatic arthritis	❑	
Allowing me to get pregnant / have children (Female respondents)	❑	
Improving my mental well-being	❑	

13. When your treatment goals / desired outcomes were set, how much influence did you have in setting these compared to your physician?

	Single Select
I had **no input** and the goals were solely **set by my physician**	❑
I had **some input** but the goals were **mainly set by my physician**	❑
My physician and I had **equal input**	❑
I had **strong input** and the goals were **mainly set by me**	❑

I had **full input** and the goals were **solely set by me**	❑

14. How regularly do you visit your physician to review your treatment success?

Weekly or more	❑
2-3 times a month	❑
Monthly	❑
Every 2-3 months	❑
Every 6 months	❑
Yearly	❑
Less often than every 12 months	❑

15. How much time does your doctor allocate to you during your regular visits?

Less than 5 minutes	❑
10-20 minutes	❑
20-30 minutes	❑
>30 minutes	❑

16. How comfortable are you in discussing the treatment(s) that work best for you with your physician?

Use a 10-point scale where "1" means not at all comfortable, and "10" means extremely comfortable

…………………. Rate (1-10)

17. Do you seek additional information other than your doctor provides?

• Yes	❑
• No	❑

18. What sources do you use to get to know more about your disease and the treatments you use for Psoriatic Arthritis?

	**Multiple select**
Doctors	❑
Pharmacists	❑
Family / friends	❑
Patient advocacy groups	❑
Internet websites / search engines	❑
Social media	❑
Product information leaflets	❑
Pharmaceutical company website	❑
Other, specify …………….	❑

19. What kind information are you interested in / searching for?

	**Multiple select**
Disease related information	❑
Treatment related information	❑
Lifestyle and dietary habits advices	❑
Home remedies	❑
Other people's experience with the disease	❑
Experts' opinion	❑
Other, specify ……………	❑

20. Thinking about when you and your physician discussed your current treatment, how satisfied or dissatisfied were you with the following aspects?

Please use a 10-point scale where "1" means not at all satisfied, and "10" means completely satisfied

	Satisfaction (1-10)
The amount of **available time for discussing treatment options** with your physician	
The **number of treatment options** your physician discussed with you	
Your **level of involvement in choosing** your current treatment	
**Overall interaction** with your physician	
Overall **education / training** received	

21. Which aspects of your disease that you would you like to discuss further with your physician?

	**Multiple select**
Impact upon my work life	❑
Impact upon my social life	❑
Impact upon my family life	❑
Impact upon how I feel / my mental/psychological wellbeing	❑
Treatment convenience	❑
Treatment tolerability	❑
Treatment safety	❑
Treatment goals / desired outcomes	❑
Other (please specify): ________________________________	❑

22. What treatment / treatments are you currently using?

Treatment	Multiple select
**Biologics & Targeted Systemic Therapies**	
Adalimumab e.g., Humira	❑
Etanercept e.g., Enbrel®	❑
Infliximab e.g., Remicade®, Remsima®	❑
Golimumab e.g., Simponi®	❑
Certolizumab Pegol e.g., Cimzia®	❑
Ustekinumab e.g., Stelara®	❑
Secukinumab e.g., Cosentyx®	❑
Ixekizumab e.g., Taltz®	❑
Guselkumab e.g., Tremfya®	❑
Risankizumab e.g., Skyrizi®	❑
Tildrakizumab e.g., Ilumya®	❑
Apremilast e.g., Otezla®	❑
Tofacitinib e.g., Xeljanz®	❑
**Topical treatment**	
Ointment/cream/gel/foam/spray	❑
**Phototherapy**	
UVA1 / PUVA / NB-UVB	❑
**Conventional Systemic Therapy**	
Cyclosporine	❑
Sulphasalazine	❑
Methotrexate (MTX)	❑
Cortisone (corticosteroids)	❑
Leflunomide	❑
Hydroxychloroquine	❑

23. How do you describe your compliance with the specific dose and timing of your medication?

	Single Select
May miss many doses per year (>5 doses)	❑
May miss few doses per year (3 to 5 doses)	❑
Rarely miss any doses (1 to 3 doses per year)	❑
Never miss any dose	❑

24. Why you may miss some doses of your treatment?

Treatment	Multiple select
Don't understand the directions correctly	❑
Misunderstand the treatment regimen	❑
Forgetfulness	❑
I feel I don't need the treatment	❑
I have other commitments conflicting with the treatment time	❑
Complex treatment regimen	❑
Psychological factor	❑
If others specify………..	❑

25. Overall, how satisfied are you with the current treatment?

Please use a 10-point scale where "1" means not at all satisfied, and "10" means completely satisfied

……………….. Satisfaction (1-10)

26. To what extent have your initial expectations for the treatment been matched by the results achieved?

Please use a 10-point scale where "1" means not at all matched, and "10" means completely matched

	Rate (1-10)
Reducing or alleviating my skin-related symptoms	
Reducing or alleviating my joint-related symptoms	
Improving my overall, everyday quality of life	
Improving my ability to socialize/interact with friends and family	
Allowing me to engage in exercise/fitness activities/sports	
Allowing me to carry out usual, daily tasks	
Improving my ability to perform at work	
Improving my disease sufficiently to allow me to have a job	
Allowing me to work full-time	
Allowing me to pursue my desired career	
Minimizing the pain I experience due to my psoriatic arthritis (taking joint and skin pain into consideration)	
Minimizing the fatigue I experience from my psoriatic arthritis	
Improving my physical functioning and mobility	
Slowing the progression of my psoriatic arthritis	
Allowing me to get pregnant / have children	
Improving my mental well-being	

DISCLAIMER: The above article has been published, as is, ahead-of-print, to provide early visibility but is not the final version. Major publication processes like copyediting, proofing, typesetting and further review are still to be done and may lead to changes in the final published version, if it is eventually published. All legal disclaimers that apply to the final published article also apply to this ahead-of-print version.
